# Pathophysiological basis of the cardiological benefits of SGLT-2 inhibitors: a narrative review

**DOI:** 10.1186/s12933-023-01855-y

**Published:** 2023-06-30

**Authors:** Cristina Panico, Benedetta Bonora, Antonella Camera, Nino Cristiano Chilelli, Giuliana Da Prato, Giuseppe Favacchio, Valeria Grancini, Veronica Resi, Maurizio Rondinelli, Emanuela Zarra, Basilio Pintaudi

**Affiliations:** 1 Department of Biomedical Sciences, Humanitas University, Pieve Emanuele-Milan, Italy; 2 IRCCS Humanitas Research Hospital, Rozzano-Milan, Italy; 3 Department of Medicine, Division of Metabolic Diseases, University of Padova, Via Giustiniani 2, Padua, 35128 Italy; 4 Diabetologia, ASST Pavia, Padua, Italy; 5 Diabetology and Internal Medicine, Hospital of Cittadella, AULSS 6 Euganea (Padua), Padua, Italy; 6 Divisione di Endocrinologia, Diabetologia e Malattie del Metabolismo, Dipartimento di Medicina, Azienda Ospedaliera Universitaria Integrata di Verona, Ospedale Maggiore, Verona, Italy; 7 U.O di Endocrinologia e Diabetologia, IRCCS Humanitas Research Hospital, Rozzano, MI Italy; 8 Endocrinology Unit, Fondazione IRCCS Ca’ Granda - Ospedale Maggiore Policlinico, Milan, Italy; 9 Diabetes Endocrine and Metabolic Diseases Unit, IRCCS Centro Cardiologico Monzino, Milan, Italy; 10 S.C. Medicina Diabetologia, Dipartimento di Continuità di Cura e Fragilità, ASST Spedali Civili, Brescia, Italy; 11 Diabetes Unit, Niguarda Hospital, Milan, Italy

**Keywords:** SGLT2 inhibitors, Type 2 diabetes, Cardiovascular, Mechanism of action, Pathophysiology, Heart failure

## Abstract

In recent years, GLP-1 receptor agonists (GLP-1RA), and SGLT-2 inhibitors (SGLT-2i) have become available, which have become valuable additions to therapy for type 2 diabetes as they are associated with low risk for hypoglycemia and cardiovascular benefits. Indeed, SGLT-2i have emerged as a promising class of agents to treat heart failure (HF). By inhibiting SGLT-2, these agents lead to excretion of glucose in urine with subsequent lowering of plasma glucose, although it is becoming clear that the observed benefits in HF cannot be explained by glucose-lowering alone. In fact, multiple mechanisms have been proposed to explain the cardiovascular and renal benefits of SGLT-2i, including hemodynamic, anti-inflammatory, anti-fibrotic, antioxidant, and metabolic effects. Herein, we review the available evidence on the pathophysiology of the cardiological benefits of SGLT-2i. In diabetic heart disease, in both clinical and animal models, the effect of SGLT-2i have been shown to improve diastolic function, which is even more evident in HF with preserved ejection fraction. The probable pathogenic mechanisms likely involve damage from free radicals, apoptosis, and inflammation, and therefore fibrosis, many of which have been shown to be improved by SGLT-2i. While the effects on systolic function in models of diabetic heart disease and HF with preserved ejection fraction is limited and contrasting, it is a key element in patients with HF and reduced ejection fraction both with and without diabetes. The significant improvement in systolic function appears to lead to subsequent structural remodeling of the heart with a reduction in left ventricle volume and a consequent reduction in pulmonary pressure. While the effects on cardiac metabolism and inflammation appear to be consolidated, greater efforts are still warranted to further define the entity to which these mechanisms contribute to the cardiovascular benefits of SGLT-2i.

## Introduction

At present there are a number of classes of pharmacological agents used for type 2 diabetes (T2D), with multiple agents available in each class [1]. In recent years, new classes of agents have become available, namely GLP-1 receptor agonists (GLP-1RA), and SGLT-2 inhibitors (SGLT-2i). These newer agents have become valuable additions to therapy for T2D as they are associated with low risk for hypoglycemia as well as cardiovascular (CV) benefits [2]. As a result, treatment approaches are changing rapidly with preference now given to agents whose benefits extend beyond glucose-lowering [2]. To aid the prescriber, comprehensive algorithms are now available for diabetes management, which emphasize personalization of glycemic targets and CV risk [2–4].

Of note, T2D is one of the main risk factors for CV diseases. It is directly associated with diabetic cardiomyopathy [5] and increases risk of ischemic cardiomyopathy and heart failure (HF) [6]. In this regard, SGLT-2i have emerged as a promising class of agents to treat HF and have a prominent role in current guidelines [7], and some of these agents have been approved for treatment of HF.

Four major CV outcome trials (CVOTs) – EMPA-REG (empagliflozin), CANVAS (canagliflozin), DECLARE-TIMI 58 (dapagliflozin), and VERTIS CV (ertugliflozin), investigated the CV benefits of SGLT-2i in patients with T2D [8–11]. Reductions in the relative risk for hospitalizations for HF of 30%, 33%, 27%, and 35%, respectively, were seen in these four trials [8–11]. In addition, a meta-analysis of CVOTs on over 34,000 patients found that SGLT-2i reduced the risk of major adverse CV events by 11%, with benefits only for patients with atherosclerotic CV disease [12].

Faced with these promising results, dedicated trials in patients with HF were initiated [13–15]. Based on these results, the EMA approved dapagliflozin for the treatment of HF with reduced and preserved ejection fraction (HFrEF, HFpEF) and empagliflozin for symptomatic chronic HF. Moreover, a class effect has been suggested based on meta-analyses [16, 17].

By inhibiting SGLT-2, SGLT-2i lead to excretion of glucose in urine with subsequent lowering of plasma glucose [18]. However, it is unlikely that the observed benefits in HF can be explained by glucose-lowering alone. In fact, multiple mechanisms have been proposed to explain the CV and renal benefits of SGLT-2i, including hemodynamic, anti-inflammatory, anti-fibrotic, antioxidant, and metabolic effects [19–21]. Furthermore, several cardiac morphofunctional parameters have been shown to be modified by this drug class, and it is possible that different mechanisms can reverse physio-pathological alterations that are typical of diabetes and HF. Herein, we review the available evidence on the pathophysiology of the cardiological benefits of SGLT-2i in different groups of patients, namely those with diabetic cardiomyopathy, HFpEF, and HFrEF.

## Materials and methods

A literature search was performed on PubMed for relevant articles and abstracts. The search string used was (“Sodium-Glucose Transporter 2 Inhibitors“[tiab] OR “Sodium-Glucose Transporter 2 Inhibitor“[tiab] OR “Sodium-Glucose Transporter 2 Inhibitors“[Mesh] OR “SGLT2-i“[tiab] OR “dapagliflozin“[tiab] OR “empagliflozin“[tiab] OR “canagliflozin“[tiab]) AND (“stroke volume“[mesh] OR “ejection fraction“[tiab] OR “Doppler“[tiab] OR “wedge“[tiab] OR “pulmonary artery pressure“[tiab] OR “end-diastolic volume“[tiab] OR “end-diastolic pressure“[tiab] OR “cardiac index“[tiab] OR “stroke volume“[tiab]) AND (mechanism*[tiab] OR pathway*[tiab]) AND (“Heart Failure“[Mesh] OR “heart failure”[tiab]). Papers were selected for inclusion in the present review according to their relevance, as judged by the authors. As a literature review, no ethics committee approval was needed.

### Effects in diabetic cardiomyopathy: animal and human studies

A number of studies have examined the effects of SGLT-2i on cardiac structure and function in animals and humans with diabetes (Table 1).

**Table 1:** Effects in diabetic cardiomyopathy: animal and human studies

Author, year	Study characteristics	Main findings
*Animal studies*		
Shih et al. 2021 [22]	Rat model with streptozotocin-induced diabetes; heart developed diabetic cardiomyopathy with fibrosis and decreased diastolic and systolic function	All of these effects were improved by treatment with dapagliflozin
Adingupu et al. 2019 [23]	Studied metabolic changes and CV function in ob/ob-/- mice	Animals given empagliflozin showed improved cardiac contractility and coronary microvascular function
*Human studies*		
Suhrs et al. 2022 [24]	Randomized, placebo-controlled cross-over study in 13 patients with T2D treated with empagliflozin 25 mg or placebo for 12 weeks	No substantial changes in CVFR were seen
Shih et al. 2021 [22]	Examined changes in left ventricular function in patients with diabetes who did not have symptomatic HF following 6 months of treatment with dapagliflozin	Both diastolic and systolic function improved despite, but no changes in LVEF
Cohen et al. [25].	Changes in cardiac structure/function examined by cardiac magnetic resonance in patients with T2D treated with empagliflozin for 6 months vs. control group	In patients receiving empagliflozin, significant reduction in left ventricular end diastolic volume with no changes in LVEF
Verma et al. 2019 [26]	Patients with T2D and CAD were randomized to empagliflozin or placebo for 6 months	Mean left ventricular mass decreased by 2.6 g/m^2^ with empagliflozin
Kayano et al. 2020 [27].	Evaluated changes in diastolic function during exercise, measuring LVFP in patients with T2D and poor glycemic control and randomized to dapagliflozin or conventional therapy for 6 months	Significant improvement diastolic function and decreases in LVFP with dapagliflozin
Rau et al. 2021 [28]	Randomized patients with T2D to empagliflozin or placebo for 3 months	No effect on LVEF, but significant improvement in diastolic function
Ikonomidis et al. [29]	Patients with T2D were randomized to insulin, liraglutide, empagliflozin, or liraglutide + empagliflozin as add-on to metformin	The effects of empagliflozin were potentiated in combination with a GLP-1 RA
Bonora et al. 2019 [30]	Used impedance cardiography to study patients with T2D and without established CV disease or HF randomized to dapagliflozin or placebo for 12 weeks	No changes in any parameter seen

CAD, coronary artery disease; CV, cardiovascular; HF, heart failure; LVEF, left ventricular ejection fraction; LVFP, left ventricular filling pressure; T2D, type 2 diabetes

#### Animal studies

The effects of dapagliflozin on myocardial function have been evaluated in a rat model with streptozotocin-induced diabetes, wherein the heart developed diabetic cardiomyopathy with pronounced fibrosis and a decline in diastolic and systolic function [22]. All of these effects were improved by treatment with dapagliflozin. Under high glucose conditions, cardiomyocytes showed significant activation of apoptosis, reactive oxygen species, and endoplasmic reticulum (ER) stress–associated proteins, which were attenuated by the coincubation with dapagliflozin.

Adingupu et al. studied metabolic changes and CV function in ob/ob-/- mice, a model for obesity and reduced glucose tolerance characterized by systolic microvascular dysfunction [23]. Compared to untreated animals, those administered empagliflozin showed improved cardiac contractility as well as coronary microvascular function as assessed by coronary flow velocity reserve (CFVR) and fractional area change.

#### Human studies

Cardiac contractility and CFVR were studied by Suhrs et al. in a randomized, placebo-controlled cross-over study in 13 patients with T2D treated with empagliflozin 25 mg or placebo for 12 weeks [24]. No substantial changes in CVFR were demonstrated, which may have been related to the short time of treatment.

Shih et al. examined changes in left ventricular function in patients with diabetes who did not have symptomatic HF following 6 months of treatment with dapagliflozin [22]. Both diastolic function and systolic function estimated with longitudinal strain improved despite no substantial changes in left ventricular ejection fraction (LVEF). Contrasting data have been obtained regarding reduction of ventricular mass. Changes in cardiac structure and function have been examined by cardiac magnetic resonance in patients with T2D treated with empagliflozin for 6 months and compared to a control group of patients with T2D not administered a SGLT-2i [25]. In patients receiving empagliflozin, a significant reduction in left ventricular end diastolic volume was seen, from a mean of 155 mL at baseline to 145 mL at 6 months, while no differences in left ventricular mass, ejection fraction, or heart rate were observed in either group. In contrast, Verma et al. found significant changes in left ventricular mass following treatment with empagliflozin [26]. In their study, 97 patients with T2D and CAD were randomized to empagliflozin or placebo for 6 months. Mean left ventricular mass decreased by 2.6 g/m^2^ with empagliflozin compared to 0.01 g/m^2^ in patients receiving placebo.

Kayano and colleagues investigated changes in diastolic function during exercise, measuring left ventricular filling pressure (LVFP) and right ventricular systolic pressure (RVSP) in 78 patients with T2D and poor glycemic control [27]. Participants were randomized to dapagliflozin or conventional add-on therapy for 6 months. Significant improvement of diastolic function was seen with decreases in both RVSP and LVFP at 6 months in patients treated with the SGLT-2i, while no changes were seen on the control group. No differences were observed in stroke volume index and cardiac index in either group.

Diastolic function has also been examined by Rau et al. who randomized 40 patients with T2D to empagliflozin or placebo for 3 months [28]. Empagliflozin had no effect on the systemic vascular resistance index, cardiac index, stroke volume index at any time point, and there was no difference in LVEF. However, empagliflozin was seen to significantly improve diastolic function (defined as reduction of early mitral inflow velocity relative to early diastolic left ventricular relaxation [E/e’]) that was significant after the first day of treatment.

Ikonomidis et al. investigated the effects of a number of glucose-lowering therapies on cardiac hemodynamic parameters [29]. Altogether, 160 patients with T2D were randomized to insulin, liraglutide, empagliflozin, or liraglutide + empagliflozin as add-on to metformin (40 patients in each group) for 12 months. At study end, perfused boundary region, a biomarker of endothelial integrity, pulse wave velocity (PWV), and systolic function estimated as global strain (longitudinal, circumferential, and radial) were improved in all subgroups of patients. However, those receiving liraglutide, empagliflozin, or the combination had a greater reduction of perfused boundary region, PWV, and systolic blood pressure compared to those receiving insulin, Global work index was improved in those receiving liraglutide alone or in combination (12.7% and 17.4%) compared with insulin or empagliflozin (3.1% and 2%). Patients receiving empagliflozin alone or in combination had a significantly greater decrease in PWV (10.1% and 13%) and central and brachial systolic blood pressure compared to insulin or liraglutide (PWV, 3.6% and 8.6%). Thus, the effects of an SGLT-2i were potentiated when used in combination with a GLP-1 RA.

Impedance cardiography is a non-invasive method that allows calculation of stroke volume, cardiac output, and other hemodynamic variables [30]. The technique has been used to study 33 patients with T2D and without established CV disease or HF who were randomized to either dapagliflozin or placebo for 12 weeks. At study end, no significant changes were seen in stroke volume, cardiac output, or cardiac index. Likewise, no changes were seen in any measures of systolic or circulatory function after treatment. The lack of effects on these parameters may be due to the short observation period and/or a sample size that is not sufficiently powered to detect differences in these measures.

### Effects in heart failure with preserved ejection fraction: animal and human studies

Animal and human studies in heart failure with preserved ejection fraction are listed in Table 2.

**Table 2:** Effects in heart failure with preserved ejection fraction: animal and human studies

Author, year	Study characteristics	Main findings
*Animal studies*		
Habibi et al. 2017 [31]	Studied diastolic function in obese female db/db mice fed chow with or without empagliflozin	Empagliflozin associated with improved left ventricular filling pressure and less interstitial fibrosis vs. control animals
Connelly et al. 2020 [43]	Pressure-volume (P-V) relationship analysis used to study changes in cardiac function in animals with experimental myocardial infarction	Empagliflozin therapy improved cardiac function independent of loading condition
Pabel et al. 2018 [33]	Performed contractility experiments with in toto-isolated systolic end-stage HF ventricular trabeculae from mice	Empagliflozin had direct pleiotropic effects on myocardium by improving diastolic stiffness and diastolic function
Lee et al. 2019 [34]	Studied cardiac hemodynamics in rats with induced non-diabetic hypertensive heart failure	Empagliflozin improved hemodynamics and attenuated cardiac fibrosis
Cappetta et al. 2020 [35]	Studied diastolic function in rats with induced non-diabetic HFpEF	Dapagliflozin improved diastolic function and had a positive effect on the myocardium
Zhang et al. 2019 [36]	Hypertension/hyperlipidemia-induced HFpEF pig model given dapagliflozin for 9 weeks	Dapagliflozin significantly diminished cardiac concentric remodeling with no changes in diastolic function
Kolijn et al. 2021 [37]	Obesity model of HFpHF in rats	Empagliflozin suppressed increased levels of ICAM-1, VCAM-1, TNF-α, and IL-6 in myocardium
Zhang et al. [39]	Mouse model of HFpEF	Expression of HMGB1 was increased in cardiac tissue and decreased by empagliflozin
*Human studies*		
Soga et al. 2021 [40]	Patients who had been administered at least 1 antidiabetic agent other than an SGLT-2i treated with dapagliflozin for 6 months	Left ventricular diastolic function ratio of (mitral inflow E to the mitral e’ annular velocities [E/e’]) significantly decreased
Tanaka et al. 2020 [41]	Patients who had been administered at least 1 antidiabetic agent other than an SGLT-2i treated with dapagliflozin for 6 months	Improvement of LV longitudinal myocardial function, which led to further improve

HF, heart failure; HFpEF, heart failure with preserved ejection fraction; LV, left ventricular

#### Animal studies

Habibi et al. reported that empagliflozin improves diastolic function in obese female db/db mice [31]. In particular, these animals were fed chow with or without empagliflozin for 5 weeks. In addition to improvements in glycemic parameters, empagliflozin was associated with improved left ventricular filling pressure (< E/E’ ratio) and less interstitial fibrosis compared to control animals. Improved diastolic function with empagliflozin was also observed in a nondiabetic model of HFpEF [32]. In addition, studies in myocardial fibers from patients and rats with diastolic HFpEF have shown that empagliflozin reduced passive stiffness of myofilaments through increases in phosphorylation of myofilament regulatory proteins [33].

In a hypertensive model of HFpEF in rats, empagliflozin was associated with significantly decreased cardiac fibrosis in atrial and ventricular tissues, and the atrial and ventricular expression of several molecules including PPARα, natriuretic peptides, and TNF-α were normalized [34]. In a similar model, rats were administered dapagliflozin for 6 weeks [35]. As monitored by echo-Doppler and heart catheterization, significant improvement in diastolic function was seen. Treatment with dapagliflozin also reduced diastolic overload of Ca^2+^ and Na^+^. Dapagliflozin further inverted endothelial activation, and reduced cardiac inflammatory markers such as NFkB and IL-6 as well as pro-fibrotic signaling via TGF-b.

A study in a hypertension/hyperlipidemia-induced HFpEF pig model, dapagliflozin administered for 9 weeks significantly diminished cardiac concentric remodeling, although no improvements in diastolic function were observed [36]. Moreover, elevations in the inflammatory cytokines IL-6 and TNF-α were seen in aortic tissue in control pigs that were inhibited by dapagliflozin. Thus, multiple benefits in the coronary epithelium were observed. In an obesity model of HFpHF in rats, empagliflozin was also found to significantly suppress increased levels of ICAM-1, VCAM-1, TNF-α, and IL-6 in myocardium [37]. In addition, higher levels of oxidative stress-dependent activation of eNOS and PKGIα oxidation were seen, which were attenuated by empagliflozin. Levels of NO, cGMP, and PKGIα in HFpEF were all increased during treatment with empagliflozin, which was associated with augmented phosphorylation of myofilament proteins. Moreover, oxidative stress and PKGIα polymerization was found to correlate with increased cardiomyocyte stiffness and diastolic dysfunction in patients with HFpEF.

Lastly, HMGB1 appears to serve as a driver of inflammation for CV disease [38]. In a mouse model of HFpEF, expression of HMGB1 was found to be increased in cardiac tissue of HFpEF mice, which is decreased by empagliflozin [39].

#### Human studies

In patients with T2D and stable HF with preserved EF, Soga et al. investigated the effect of dapagliflozin on left ventricular diastolic function [40]. Patients who had been administered at least 1 antidiabetic agent other than an SGLT-2i initiated treatment with dapagliflozin for 6 months. Left ventricular diastolic function ratio of (mitral inflow E to the mitral e’ annular velocities [E/e’]), significantly decreased from 9.3 to 8.5 after 6 months. Multivariate logistic regression analysis found that that dyslipidemia was the only independent factor associated with improvement in E/e’. Moreover, the change in E/e’ after 6 months for patients with dyslipidemia was significantly larger than that for patients without dyslipidemia. In the same patients, improvements in global longitudinal strain were also observed [41].

### Effects in heart failure with reduced ejection fraction: animal and human studies

#### Animal studies

Several animal studies have attempted to shed additional light on the mechanisms behind the cardiac benefits of SGLT-2i in patients with HF (Table 3). In a diabetes mice model of ischemic cardiomyopathy, Ideishi et al. treated animals with vehicle, empagliflozin, linagliptin, and empagliflozin + linagliptin before inducing myocardial ischemia for 30 min [42]. Combination therapy was found to significantly preserve cardiac systolic function as monitored by left ventricular volume at the peak left ventricular ejection rate. Combination therapy also appeared to have an anti-fibrotic effect compared to the control group that was not dependent on blood glucose levels.

**Table 3:** Effects in heart failure with reduced ejection fraction: animal and human studies

Author, year	Study characteristics	Main findings
*Animal studies*		
Ideishi et al. 2021 [42]	Streptozotocin-induced diabetic mice were divided into control, empagliflozin, linagliptin, and combination groups and treated for 7 days	Combination therapy with linagliptin and empagliflozin preserved cardiac systolic function
Connelly et al. 2020 [43]	Non-diabetic rats with induced MI of the LV randomized to vehicle or empagliflozin	Empagliflozin improved cardiac function independent of loading conditions
	Non-diabetic rats with induced MI randomized to empagliflozin or control chow for 2 weeks	Empagliflozin improved cardiac function, remodeling, cardiac metabolism
Daud et al. 2021 [45]	Non-diabetic rats with induced MI randomized to empagliflozin or control for 4 weeks	Empagliflozin reduced myocardial fibrosis and inhibited the TGF-beta1/Smad3 fibrotic pathway
Baker et al. 2019 [46]	Lean swine received canagliflozin or placebo 24 h before temporary occlusion of the circumflex coronary artery	Canagliflozin preserved cardiac contractile function and efficiency and provided ischemia protection
Goerg et al. 2021 [47]	Studied effects of empagliflozin on cardiac function in normoglycemic rats for 7 days followed by experimental MI	Empagliflozin increased cardiac output, stroke volume, and fractional shortening and after MI improved global longitudinal strain
Ren et al. 2021 [48]	Male randomized into control, empagliflozin, sunitinib, or sunitinib + empagliflozin for 28 days	Mice treated with suninitib showed decreased LVEF compared to controls that was attenuated with empagliflozin
Gong et al. 2021 [49]	Rat cardiac cells exposed to hypoxia/reoxygenation to simulate an ischemia/reperfusion (I/R) injury	Co-administration of rosuvastatin and dapagliflozin significantly enhanced cell viability and upregulated expression of p-PI3K, p-Akt, p-mTOR, and Bcl-2.
Lin et al. 2021 [50]	Rats with mitral regurgitation induced left heart dilatation and functional decline	Dapagliflozin partially restored LVEF and significantly diminished cardiac fibrosis and apoptosis
Shi et al. 2019 [51]	Transverse aortic constriction induced cardiac remodeling in mice then treated with dapagliflozin	Dapagliflozin improved cardiac systolic function, and inhibited myocardial fibrosis and cardiomyocyte apoptosis
Yerra et al. 2022 [52]	Transverse aortic constriction induced cardiac remodeling in mice then treated with empagliflozin	Empagliflozin attenuated LV enlargement in mice
Chen et al. [53]	Model of diabetes using male BTBR ob/ob mice; dapagliflozin, ticagrelor, or both administered for 12 weeks	Both agents improved left ventricular end-systolic and end-diastolic volumes as well as LVEF
Byrne et al. 2020 [54]	Rodent model of HF administered empagliflozin	Empagliflozin associated with reduced cardiac inflammation via blunting activation of the NLRP3 inflammasome in a Ca(2+)-dependent manner
Santos-Gallego et al. 2019 [55]	Heart failure induced in nondiabetic pigs that were randomized to empagliflozin or placebo for 2 months	Empagliflozin ameliorated adverse cardiac remodeling and HF and improved myocardial energetics
Li et al., 2021 [56]	Mice subjected to sham surgery or transverse aortic constriction and after 2 weeks given empagliflozin or vehicle was for 4 weeks.	Empagliflozin increased glucose and fatty acid oxidation in failing hearts, while reducing glycolysis
*Human studies*		
Thirunavukarasu et al. [57]	Patients with T2D underwent cardiac magnetic resonance and (31)P-MRS scans before and after 12 weeks of empagliflozin	Improvements seen in LVEF, global longitudinal strain, and mean myocardial cell volume
Chan et al. [58]	Compare changes in echocardiographic parameters in patients with T2D receiving SGLT2i with a different baseline LV ejection fraction (LVEF).	SGLT2i associated with an improvement in LV systolic function in patients with T2DM with reduced and moderately reduced LVEF
Lee et al. 2021 [59]	Randomized patients with T2D/prediabetes and HFrEF to empagliflozin or placebo for 36 weeks	Empagliflozin reduced LV volumes
Ilyas et al. 2021 [60]	Investigated the effects of 2 weeks of therapy with dapagliflozin in patients with T2D and HFrEF	No functional effects on cardiac function were observed
Santos-Gallego et al. 2021 [61].	Randomized HFrEF patients without diabetes to empagliflozin or placebo for 6 months	Empagliflozin significantly improved LV volumes, LV mass, LV systolic function, and functional capacity
Omar et al. 2021 [62]	Post hoc analysis of the randomized Empire HF trial in patients with HFrEF treated with empagliflozin or placebo for 12 weeks	Empagliflozin associated with modest reductions in LV and left atrial volumes with no association with ejection fraction
Hwang et al. 2020 [63]	Evaluated patients with diabetic patients with HF by echocardiography before, and 6 to 24 months after the initiation of SGLT2i	SGLT2i improved cardiac function, regardless of the presence of HF, but the improvements were more prominent in HF patients, especially in those with HFrEF
Maragkoudakis et al. 2021 [64]	Prospective cohort study of symptomatic HF patients with EF < 35% who had dapagliflozin added to therapy	Dapagliflozin improved both systolic and diastolic function
Omar et al. 2020 [65]	Randomized patients with HFrEF to empagliflozin or placebo once daily for 12 weeks	Empagliflozin significantly reduced pulmonary capillary wedge pressure
Mullens et al. 2020 [66]	Investigated effects of dapagliflozin after 7 days in consecutive HFrEF with elevated pulmonary artery pressure	Dapagliflozin reduced pulmonary artery pressure
Requena-Ibanez et al. [67].	Patients without diabetes and HFrEF underwent cardiac magnetic resonance at baseline and after 6 months of empagliflozin	Empagliflozin improved adiposity, interstitial myocardial fibrosis, aortic stiffness, and inflammatory markers

HF, heart failure; HFrEF, heart failure with reduced ejection fraction; LV, left ventricle; LVEF, left ventricular ejection fraction; MI, myocardial infarction; T2D, type 2 diabetes

SGLT-2i inhibitors appear to be of benefit when given both before and after MI. For example, in another model of HFrEF due to ischemic cardiomyopathy, pressure-volume (P-V) relationship analysis was used to examine changes in intrinsic cardiac function in animals with experimental MI [43]. Following confirmation of infarct size at 1 week post-infarction, diabetic mice received either empagliflozin or vehicle for 6 weeks. Load-insensitive measures of cardiac function were found to be improved with empagliflozin compared to vehicle. In addition, load-independent measures of cardiac contractility, preload recruitable stroke work, and end-systolic pressure volume relationship were all greater in animals randomized to empagliflozin. There was also a reduction in left ventricular end-diastolic pressure with empagliflozin.

The effects of SGLT-2i have also been evaluated in non-diabetic experimental models. In one study, non-diabetic rats underwent sham surgery or permanent coronary artery ligation to induce MI [44]. The animals received empagliflozin-containing chow (2 weeks before or 2 weeks after surgery, or control chow). While empagliflozin had no effect on the size of the MI, the LVEF was significantly higher in both groups receiving empagliflozin either 2 weeks before or 2 weeks after surgery compared to vehicle. Empagliflozin also reduced cardiomyocyte hypertrophy, and decreased both interstitial fibrosis and oxidative stress in the myocardium. A number of metabolic changes were observed that were associated with an increase in cardiac ATP production. These included an increase in circulating ketone levels and myocardial expression of the ketone body transporter and key ketogenic enzymes.

Mechanistic insights have also been provided. In a nondiabetic rat model of ischemic HF, animals were administered, or not, empagliflozin immediately after induction of MI [45]. In animals treated with empagliflozin, collagen deposition was found to be significantly lower in both the scar and remote cardiac areas. Expression of TGF-β1 and Smad3 were both decreased by empagliflozin. Thus, empagliflozin decreased myocardial fibrosis possibly through inhibition of the TGF-β1/Smad3 pathway.

Cardiac contractile function and myocardial substrate utilization has been assessed in lean swine that received canagliflozin or placebo 24 h before temporary occlusion of the circumflex coronary artery. Invasive study protocol was performed at baseline, during.

Coronary occlusion and during 2 h of reperfusion [46]. At the onset of ischemia, canagliflozin was associated with large increases in left ventricular end-diastolic and systolic volumes which during reperfusion returned to baseline levels. Canagliflozin administration further increased end-diastolic volume, stroke volume, and stroke work vs. control animals during ischemia, and augmented cardiac work efficiency during ischemia. It was concluded that canagliflozin provides protection from ischemia that is independent of changes in utilization of myocardial substrate given that no changes seen in myocardial uptake of glucose, lactate, ketones, or free fatty acid were observed.

In an investigation in healthy normoglycemic rats, at 30 min after the administration of empagliflozin, increases in ventricular systolic pressure, mean pressure, and the max dP/dt were observed [47]. Empagliflozin given for one week was found to increase cardiac output, stroke volume, and fractional shortening. At 7 days after induction of MI through ligation of the left coronary artery, empagliflozin improved global longitudinal strain compared to vehicle (-21.0% vs. -16.6%, respectively). Analysis of infarcted tissues showed that empagliflozin decreased the expression of matrix metalloproteinase 9 (MMP9) and also regulated the cardiac transporters SERCA2a and NHE1. In vitro, empagliflozin decreased the activity of MMP2 and MMP9 and also prevented apoptosis. mTOR signaling has also been implicated in the cardioprotective effects of SGLT-2i.

mTOR has also been implicated in a mouse model of cardiac dysfunction induced by sunitinib that was used to study the protective effects of empagliflozin [48]. Mice treated with sunitinib showed decreased LVEF compared to control animals (50.2% vs. 84.9%, respectively) that was attenuated with administration of empagliflozin (76.18%). In cardiomyocytes studied in vitro, it was found that sunitinib led to cell death and dysfunction of AMPK-mTOR signaling that were reversed by the addition of empagliflozin. The PI3K/AKt/mTOR signaling pathway was also implicated in another in vitro study in which H9C2 cells were exposed to hypoxia/reoxygenation to simulate ischemia/reperfusion injury [49]. Co-administration of rosuvastatin and dapagliflozin not only significantly enhanced cell viability, but upregulated the expression of p-PI3K, p-Akt, p-mTOR, and Bcl-2.

In a model in rats in which mitral regurgitation (MR) induced HFrEF, animals were given no treatment or dapagliflozin for 6 weeks [50]. Dapagliflozin partially rescued MR-induced dysfunction that included partial restoration of LVEF and the end-systolic pressure volume relationship. Animals administered dapagliflozin also showed significantly diminished cardiac fibrosis, cardiac apoptosis, and expression of stress-associated proteins in the endoplasmic reticulum.

In another animal model of non-ischemic HFrEF, transverse aortic constriction (TAC) was used to induce cardiac remodeling [51]. Dapagliflozin was then administered to some of the animals for 4 weeks. Compared to those not receiving dapagliflozin, treatment with the SGLT-2i decreased myocardial hypertrophy, cardiomyocyte apoptosis, perivascular and myocardial interstitial fibrosis. Dapagliflozin also inhibited phosphorylation of the P38 and JNK proteins and inhibited phosphorylation of FoxO1, which regulate cellular pathways involved in the development of cardiac failure, including fibrosis, apoptosis, inflammatory responses, and cell proliferation, in untreated mice that was reversed by administration of dapagliflozin.

Inflammation has been clearly linked in the CV benefits of SGLT-2i. In the same model of TAC-induced HFrEF, treatment with inhibitor empagliflozin confirmed the effects on lessened left ventricular enlargement [52]. Furthermore, empagliflozin reduced the expression of Tnfrsf12a, a TNF superfamily receptor with proinflammatory, and prohypertrophic effects.

In an animal model of diabetes using male BTBR ob/ob mice, dapagliflozin, ticagrelor, or the combination for were administered for 12 weeks, both agents decreased progression of diabetic cardiomyopathy as shown by amelioration in left ventricular end-systolic and end-diastolic volumes as well as LVEF [53]. Both drugs also diminished activation of the NOD-like receptor 3 inflammasome. Compared to controls, significant decreases myocardial TNF-α and IL-6 were seen with both drugs alone or in combination. In nondiabetic mice with established HFrEF, Byrne et al. reported that the empagliflozin diminished activation of the NLRP3 inflammasome [54]. In addition, the same effects on the NLRP3 inflammasome were also observed in animals with HFpEF. Since a calcium ionophore blocked the ability of empagliflozin to reduce inflammation, it was suggested that empagliflozin exerts its benefit in a calcium dependent manner.

Lastly, changes in myocardial energetics were also observed in a nondiabetic swine model of HFrEF [55]. In contrast to control animals, pigs treated with empagliflozin showed increased uptake of ketone bodies, free fatty acids, and branched chain amino acids instead of glucose, as well as enhanced expression of the enzymes used for their metabolism. Thus, increases in myocardial ATP were observed together with improved myocardial work efficiency. In the same model of TAC-induced HFrEF, Li et al. reported that empagliflozin increased glucose and fatty acid oxidation in failing hearts, while reducing glycolysis [56]. This attenuated adverse cardiac remodeling and progression of heart failure reestablishing activation of adenosine monophosphate-activated protein kinase.

#### Human studies

SGLT-2i have been extensively studied in patients with HFrEF with or without diabetes. Cardiac energetics have been shown to be improved by SGLT-2i in patients with T2D [57]. After treatment, improvements were observed in LVEF, global longitudinal strain, and mean myocardial cell volume. In addition, empagliflozin was associated with significant improvement in the phosphocreatine-to-ATP ratio, from 1.52 to 1.76. The authors suggested that empagliflozin gives rise to a “cardiac energy-deficient” state that also serves to help reverse myocardial cellular remodeling and improve cardiac function.

Chan et al. published the results of a study in which they enrolled 665, 119, and 132 patients with T2D and preserved (≥ 50%), moderately reduced (40–50%), and reduced baseline LVEF (< 40%) who were receiving with SGLT-2i [58]. In addition, 212 patients receiving a DPP-4i were enrolled. After a median of 230 days, in those with reduced LVEF and treated with a SGLT-2i, LVEF improved from 29.4 to 42.2% and decrease in left ventricle end systolic volume (LVESV) decreased from 133.2 mL to 117.4 mL. In those with moderately reduced LVEF, the LVEF improved from 44.8 to 49.7%, while LVESV decreased from 90.7 mL to 80.0 mL. In contrast, in patients with preserved LVEF, there was no improvement in LVEF or LVESV following treatment with a SGLT-2i. In patients who had impaired LVEF (< 50%) at baseline and receiving a DPP-4i, there were no changes LVEF or LVESV. Thus, LV systolic function improved in patients with T2D and severe and moderately reduced LVEF after treatment with an SGLT-2i.

The SUGAR-DM-HF trial randomized 105 patients with T2D/prediabetes and HFrEF (77.1% with NYHA II and 22.9% with NYHA III) to empagliflozin or placebo and followed for 36 weeks [59]. In patients treated with empagliflozin, favorable reverse LV remodeling was observed: the LV end-systolic volume index was significantly reduced by 6.0 mL/m^2^ compared to placebo, and the LV end-diastolic volume index by 8.2 mL/m^2^ (P = 0.0042). No difference was seen in LV global longitudinal strain.

Ilyas et al. investigated the effects of two weeks of therapy with dapagliflozin in 19 patients with T2D and HFrEF in a placebo-controlled crossover trial [60]. While reductions in blood pressure were seen as expected, no functional effects on cardiac function were observed considering chamber size, ventricular systolic function, and filling pressure. However, it must be highlighted that the time on the SGLT-2i was limited to two weeks.

In patients without diabetes and HFrEF, Santos-Gallego carried out a study in which 84 participants were randomized to empagliflozin or placebo for 6 months [61]. Empagliflozin was associated with a significant decrease in LV end-diastolic volume compared to placebo (-25.1 mL vs. -1.5 mL, respectively) and LV end-systolic volume (-26.6 mL vs. -0.5 mL, respectively). Moreover, compared to placebo significant reduction in LV mass was seen (-17.8 g vs. 4.1 g, respectively) as well as improvement in LVEF (6.0 vs. -0.1, respectively). Compared to placebo, empagliflozin also improved peak O_2_ consumption, oxygen uptake efficiency slope, and the 6-min walk test.

In a post hoc analysis of the randomized Empire HF trial involving 190 patients with HFrEF (12.6% with T2D), empagliflozin was associated with significantly reduced LVESV (-4.3 mL/m^2^), left ventricular end-diastolic volume (-5.5 mL/m^2^), and left atrial volume (-2.5 mL/m^2^) compared with placebo after 12 weeks, although no change in LVEF was seen [62]. As noted by the authors, further study is warranted to examine the effects of empagliflozin beyond 12 weeks. Considering longer treatment times, Hwang et al. followed 304 patients with T2D for a median of 13 months [63]. Changes in cardiac function were investigated in 4 groups of patients: group 1 (without HF nor SGLT-2i), group 2 (without HF and received SGLT-2i), group 3 (with HF but no SGLT-2i), and group 4 (with HF and received SGLT-2i). Patients with HF being treated with an SGLT-2i showed a significant decrease in left ventricular end-diastolic dimension in addition to a significant improvement in the LVEF. LV mass index and diastolic parameters were also improved in HF patients and administered an SGLT-2i. The improvements in cardiac function seen in patients receiving an SGLT-2i were less prominent in patients without HF and in those with HFpEF. The effects of dapagliflozin on echocardiographic parameters have also been examined in 30 patients with diabetes and HFrEF [64]. The addition of dapagliflozin improved both systolic and diastolic function.

The effects of empagliflozin on the ratio of pulmonary capillary wedge pressure (PCWP) to cardiac index (CI) at peak exercise have been studied in 70 patients with HFrEF (mean LVEF 26%, 17% with T2D) [65]. While no significant effect on peak PCWP/CI was observed, PCWP was significantly reduced (-2.40 mm Hg) without improvement of CI. Reductions were seen in patients with and without T2D. Mullens et al. investigated the effects of dapagliflozin on systolic pulmonary artery pressure (PAP) with an implantable system that provides real-time remote monitoring of pressure [66]. Changes in PAP were monitored in 9 patients with HFrEF before and after 7 days of therapy with dapagliflozin. Mean PAP decreased from 42 mmHg to 38 mmHg during therapy with dapagliflozin. Of note, the reduction in PAP occurred within the first 2 days of treatment and remained stable throughout the entire study period.

A secondary analysis of patients enrolled in the EMPA-TROPISM trial investigating empagliflozin in patients without diabetes and HFrEF evaluated the effect of SGLT2i in cardiac remodeling [67]. Empagliflozin treatment was significantly associated with a reduction in epicardial adipose tissue (-5.14 mL vs. -0.75 mL), interstitial myocardial fibrosis estimated by T1 mapping (-1.25% vs. 0.24%) and cardiomyocyte volume (-11.08 mL vs. 0.80 mL). Proteomic analysis furthermore demonstrated that empagliflozin was associated with a significant reduction in several markers of inflammation, including E-selectin and TNFRSF10C.

## Discussion

### Effects in patients with diabetic cardiomyopathy

Diabetes mellitus, even in the absence of other risk factors such as arterial hypertension, obesity, and coronary artery diseaseis known to be associated with high risk for CV complications. The main cardiac morphofunctional alterations associated with diabetes are hypertrophy and diastolic dysfunction [68]. A number of mechanisms have been proposed to explain these effects, and many have been implicated to rationalize the cardiac benefits of SGLT-2i in patients with diabetes as seen herein.

In a study of the effects of SGLT-2i in patients with or without established CV disease no significant changes in cardiac stroke volume of output were evident [30]. Furthermore, although the number of studies analyzing systolic function in patients with diabetes is limited, SGLT-2i would not seem to directly affect this parameter [24, 30].

In contrast, diastolic function does appear to be directly influenced by SGLT-2i [22, 25, 28]. In addition, significant decreases in left ventricular mass were documented following treatment with empagliflozin [26]. This is potentially relevant considering that increased left ventricular mass and diastolic dysfunction have both been associated with associated with endothelial dysfunction [69]. An animal study documented that empagliflozin improved coronary microvascular dysfunction [23], even if no substantial changes were observed in a study in humans considering CVFR, although the treatment period may have been too short to expect any significant changes [24]. It is thus possible that SGLT-2i, by improving endothelial dysfunction, can inhibit negative cardiac remodeling and thus improve diastolic function.

### Effects in heart failure with preserved EF

The benefits of SGLT-2i have also been confirmed in the context of HFpEF both with and without diabetes. Animal models have largely confirmed these findings with improvement in LV diastolic function in animals with and without diabetes [31, 32]. Similar to studies in HFrEF, benefits in several cardiac inflammatory markers have been observed during administration of an SGLT-2i in HFpEF [34–39]. SGLT-2i thus appear to regulate inflammatory processes, which are a known pathological element in HFpEF, and may also reduce fibrosis [70]. Among the mechanisms involved in the development of HFpEF, metabolic dysfunction and inflammation are now known to have major roles [71]. Metabolic alterations and oxidative stress are capable of activating low-grade inflammation which in turn activates pathways involved in hypertrophy and fibrosis and therefore diastolic dysfunction. SGLT-2i have been shown to modulate several inflammatory pathways by reducing the level of circulating cytokines and reduce oxidative stress [34, 36–39]. Even if limited, studies in patients with diabetes and HFpEF suggest that diastolic function is improved by administration of an SGLT-2i [40, 41].

### Effects in heart failure with reduced EF

Diabetes and HFrEF are risk factors for each other and can bidirectionally independently worsen outcomes. Indeed, diabetes is a common concurrent diagnosis with HFrEF and increases the risk of HF by two- to four-fold [72]. At the same time, patients with HFrEF and diabetes have worse prognosis compared to those without diabetes [73]. SGLT-2i have been shown to significantly reduce MACE in patients with HFrEF and at the same time to reverse the most important morphofunctional alterations typical of HFrEF [74].

SGLT-2i appear to be associated with positive LV remodeling and with improvement in the LVEF along with reduced end-systolic volume [58, 59], although such changes have not been consistently seen across studies [62]. In this regard, it should be highlighted that the duration of therapy with an SGLT-2i was not consistent in these various analyses, which may help to explain some of the apparent discrepancies reported such as the 2-week study by Ilyas et al. [60].

Metabolic changes have also been noted in addition to improvement in LVEF, which may lead to benefits in cardiomyocyte contractility [57].

### Effects in HFrEF without diabetes

The effects of SGLT-2i have been more amply demonstrated in the context of HFrEF in the absence of diabetes. SGLT-2i have been shown to improve systolic function [61, 63] and myocardial oxygen consumption [61] and reduce LV mass [61, 62]. They have also been demonstrated to reduce PCWP.

Considering cardiac hemodynamics, SGLT-2i seem to be associated with improvements in systolic function, and as a consequence pulmonary hypertension, that appear to be rapid, in contrast to the effects on LVEF remodeling and mass [65, 66]. SGLT-2i have been further shown to prevent post-infarct cardiac remodeling through reduction of end-diastolic pressure [43] and improve LVEF, along with reduced cardiomyocyte hypertrophy and improved cardiac ATP production [44–47, 49–51]. These effects may possibly be due, at least in part, to improvement in cardiac energetics by increasing ketone bodies and promoting beta-oxidation with consequent improvements in cardiomyocyte contractility [55–57].

## Conclusions

Large CVOTs initially confirmed the CV benefits of SGLT-2i in patients with T2D [8–11]. However, the observed CV benefits cannot be explained solely by the effects of SGLT-2i on glycemic control and many studies have been carried out to better understand their mechanism of action. The present review aimed to focus on the direct effects of SGLT-2i on cardiac structure and function in different pathological contexts, and highlights the pleiotropic effects of this class of drugs.

In diabetic heart disease, in both clinical and animal models, the effect of SGLT-2i have been shown to improve diastolic function, which is even more evident in HFpEF where it represents a key pathogenetic element. The probable pathogenic mechanisms likely involve damage from free radicals, apoptosis, and inflammation, and therefore fibrosis, many of which have been shown to be improved by SGLT-2i. While the effects on systolic function in models of diabetic heart disease and HFpEF is limited and contrasting, it is a key aspect in HFrEF, both with and without diabetes. The significant improvement in systolic function appears to lead to subsequent structural remodeling of the heart with a reduction in the LV volume and a consequent reduction in pulmonary pressure [41, 43, 58, 59, 61, 63, 65, 66, 75]. The effects on cardiac metabolism and inflammation appear to be consolidated, although greater study is needed to further define the entity to which these mechanisms contribute to the CV benefits of SGLT-2i.

## Data Availability

Not applicable.

## Abbreviations

CV: Cardiovascular
CVOTs: Cardiovascular outcome trials
ER: Endoplasmic reticulum
GLP-1RA: GLP-1 receptor agonists
HF: Heart failure
HFpEF: Heart failure with preserved ejection fraction
HFrEF: Heart failure with reduced ejection fraction
LFEF: Left ventricular ejection fraction
LV: Left ventricle
LVFP: Left ventricular filling pressure
PWV: Pulse wave velocity
RSVP: Right ventricular systolic pressure
SGLT-2i: SGLT-2 inhibitors
T2D: Type 2 diabetes
